# Reasons for canakinumab initiation among patients with periodic fever syndromes: a retrospective medical chart review from the United States

**DOI:** 10.1186/s12969-021-00605-2

**Published:** 2021-09-14

**Authors:** Peter Hur, Kathleen G. Lomax, Raluca Ionescu-Ittu, Ameur M. Manceur, Jipan Xie, Jordan Cammarota, Raju Gautam, Navneet Sanghera, Nina Kim, Alexei A. Grom

**Affiliations:** 1 Novartis Pharmaceuticals Corporation, East Hanover, NJ USA; 2 Analysis Group, Inc, Montreal, QC Canada; 3 Analysis Group, Inc, Los Angeles, CA USA; 4 Analysis Group, Inc, Washington, DC, USA; 5 Novartis Healthcare Pvt. Ltd, Hyderabad, India; 6 Baylor Scott and White Medical Center Temple, Texas and University of Texas, Austin, Texas USA; 7 Division of Pediatric Rheumatology, Children’s Hospital Medical Center, MLC 4010, 3333 Burnet Avenue, Cincinnati, OH 45229 USA

**Keywords:** Canakinumab, Cryopyrin-associated periodic fever syndrome, Familial Mediterranean fever, Hyperimmunoglobulin D syndrome, Mevalonate kinase deficiency, Real world, Retrospective review, TNF receptor-associated periodic fever syndrome

## Abstract

**Background:**

Although canakinumab has demonstrated efficacy in multiple trials in patients with periodic fever syndromes (PFS), the evidence on initiation of canakinumab among PFS patients in real world setting is not well understood. We aimed to characterize the reasons for canakinumab initiation among patients with PFS, specifically, cryopyrin-associated periodic syndrome (CAPS), hyperimmunoglobulin D syndrome/mevalonate kinase deficiency (HIDS/MKD), TNF receptor-associated periodic syndrome (TRAPS) and familial Mediterranean fever (FMF).

**Methods:**

Physicians retrospectively reviewed the medical charts of PFS patients prescribed canakinumab between 2016 and 2018. Information collected included patient clinical characteristics, reasons for previous treatment discontinuation and canakinumab initiation. The results were summarized for overall patients, and by children (< 18 years) and adults and by subtype of PFS.

**Results:**

Fifty-eight physicians in the US (rheumatologists, 44.8 %; allergists/immunologists, 29.3 %; dermatologists, 25.9 %) abstracted information for 147 patients (children, 46.3 %; males, 57.1 %; CAPS, 36.7 %; TRAPS, 26.5 %; FMF, 26.5 %; HIDS/MKD, 6.8 %; Mixed, 3.4 %). Overall, most patients (90.5 %) received treatment directly preceding canakinumab (NSAIDs, 27.8 % [40.0 % in HIDS/MKD]; anakinra, 24.1 % [32.7 % in CAPS]; colchicine, 21.8 % [35.9 % in FMF]), which were discontinued due to lack of efficacy/effectiveness (39.5 %) and availability of a new treatment (36.1 %). The common reasons for canakinumab initiation were physician perceived efficacy/effectiveness (81.0 %; children, 75.0 %; adults, 86.1 %), lack of response to previous treatment (40.8 %; children, 38.2 %; adults, 43.0 %) and favorable safety profile/tolerability (40.1 %; children, 42.6 %; adults, 38.0 %). Within subtypes, efficacy/effectiveness was the most stated reason for canakinumab initiation in HIDS/MKD (90.9 %), lack of response to previous treatment in FMF (52.4 %) and convenience of administration/dosing in CAPS (27.1 %).

**Conclusions:**

This study provided insights into how canakinumab is initiated in US clinical practice among PFS patients, with physician perceived efficacy/effectiveness of canakinumab, lack of response to previous treatment and favorable safety profile/tolerability of canakinumab being the dominant reasons for canakinumab initiation in all patients and in children and adults and PFS subtypes. Notably, the favorable safety profile/tolerability of canakinumab was more often the reason for initiation among children versus adults.

**Supplementary Information:**

The online version contains supplementary material available at 10.1186/s12969-021-00605-2.

## Background

Periodic fever syndromes (PFS) comprise a group of rare auto-inflammatory diseases such as cryopyrin-associated periodic syndromes (CAPS), hyperimmunoglobulin D syndrome/mevalonate kinase deficiency (HIDS/MKD), tumor necrosis factor receptor-associated periodic syndrome (TRAPS), and familial Mediterranean fever (FMF). CAPS includes three usually distinct, rare, inherited inflammatory disorders which vary in severity from the mild manifestation of familial cold auto-inflammatory syndrome (FCAS), moderate severity manifestation of Muckle-Wells syndrome (MWS), to the most severe form of chronic infantile neurological cutaneous and articular syndrome (CINCA), which is also called neonatal-onset multisystem inflammatory disease (NOMID) [1–4].

Fever is the main manifestation of these auto-inflammatory diseases, although patients may also experience many other signs or symptoms including abdominal, joint, or chest pain, and skin rashes [5]. The onset of PFS symptoms usually occurs in the first years of life; however, a proportion of patients may present with first symptoms in their twenties or thirties [6]. The prevalence (per million) of CAPS is generally between 1 and 3 persons, TRAPS approximately 1–2 persons and HIDS/MKD is 1–6 individuals [1, 7, 8]. FMF is the most common of the PFS and is more prevalent amongst people of Mediterranean and Middle Eastern descent, typically Sephardic Jews, Turks, Arabs and Armenians, but is not restricted to these ancestries [9]. PFS cause uncontrolled systemic inflammation that may lead to irreversible organ damage. If untreated, FMF and TRAPS patients may also develop renal amyloidosis that can cause loss of function in the kidneys [9].

The primary goal in the management of PFS is to control the disease symptoms by suppressing inflammation. The conventional treatments such as oral corticosteroids (OCS), non-steroidal anti-inflammatory drugs (NSAIDs), and methotrexate have long been used and only provide symptomatic relief. Colchicine is the mainstay of FMF treatment, and its regular use prevents symptoms/attacks in many patients and also decreases the long-term risk of amyloidosis [10, 11]. However, colchicine is either ineffective or associated with unacceptable side effects in 5–10 % of patients with FMF [10]. Biological therapies are often started if the PFS is not controlled by conventional treatments. In the United States (US), few biological therapies are approved for PFS indications, including anti-interleukin (IL)-1 agents such as anakinra and rilonacept (both for CAPS only) and canakinumab [12–14].

Canakinumab is a fully human monoclonal antibody of the class IgG1 that acts specifically against IL-1β. Canakinumab is currently approved in the US for adult and pediatric patients with PFS, including CAPS (children 4 years of age and older), TRAPS, HIDS/MKD and FMF. Canakinumab is recently approved for patients with Still’s disease (including systemic juvenile idiopathic arthritis patients aged 2 years and older, and adult-onset still’s disease) [13]. Clinical trials have demonstrated strong efficacy of canakinumab in the treatment of patients with PFS [5, 15]. However, the evidence on reasons for the initiation of canakinumab among patients with PFS in the real world setting in US clinical practice is limited. The objective of this study was to characterize the clinical and treatment profiles of patients with PFS who were prescribed canakinumab and the physician reasons for initiating canakinumab in US clinical practice.

## Materials and methods

### Study design

This was a retrospective medical chart review to collect information on patients with PFS treated with canakinumab in the US. Physicians specializing in allergy/immunology, dermatology, or rheumatology (with adult or pediatric subspecialty) were invited through email by an external vendor, which has the largest panel of medical specialists in the US. Physicians in this panel cover all US states and have similar age, gender, and practice type distribution as American Medical Association (AMA)-registered physicians. Physicians were eligible if they had personally prescribed canakinumab for at least one patient with a PFS and agreed to extract the patient information from the medical chart in an online case report form (CRF).

### Patient selection

Patients were included if they: (a) initiated canakinumab therapy for the treatment of PFS in the past 24 months from the CRF date, (b) did not initiate canakinumab in a clinical trial, and (c) had medical records including disease characteristics and treatment history at the index date which were accessible to the participating physicians. Index date was defined as the date of the first canakinumab prescription in the patient’s medical chart.

### Data collection

Data were collected through a panel-based chart review in a double-blind manner so that the identities of participating physicians and sponsor were unknown to each other. For data extraction, it was not necessary for the patient to be currently under the reporting physician’s care as long as the physician had access to the patient’s medical chart at the time of canakinumab initiation. In the medical chart review studies, the chart extraction is usually done in one setting. Physicians prepared a list of patients who met the study eligibility criteria and were asked to randomly select 1 − 10 patients. Patient-level information collected in online CRFs comprised patient demographics, disease diagnosis, clinical characteristics at the time of canakinumab initiation, history of previous treatments and reasons for their discontinuation, prescribing pattern of canakinumab, and the reasons for canakinumab initiation. Physician-level information included age, gender, primary medical specialty and primary subspecialty, type and region of practice, and years in the practice. An external vendor collected the physician responses in a common database and data were transferred securely to the authors for analysis. No patient identifying information was collected in the CRFs and the authors did not have access to information that identifies individual patients or physicians. Ethics approval was obtained from the New England Independent Review Board (NEIRB, #120,180,302) prior to the initiation of data collection.

### Statistical methods

Descriptive statistics were reported as frequency and/or percentages for categorical variables and means and standard deviations (SD) for continuous variables. Physicians were not allowed to leave any question unanswered and “unknown” response options were included in the answer options where applicable; thus, occurrence of missing values was minimal and not imputed. Multiple steps were taken to ensure data accuracy of the completed CRFs including consistency checks, range checks, and other electronic and manual verification of data in CRFs. Results were summarized for all patients with PFS and stratified into pediatric patients (i.e., less than 18 years) and adult patients. A sub-group analysis was also performed according to the subtype of PFS (CAPS, HIDS/MKD, TRAPS, or FMF). For the subgroup analyses, patients with mixed PFS subtypes were included in all relevant PFS subgroups.

## Results

Fifty-eight physicians participated with an overall response rate of less than 15 % (8–13 % depending on the specialty). They contributed 147 medical charts of PFS patients (68 [46.3 %] children and 79 [53.7 %] adults), with an average 3.6 charts per physician.

### Physician characteristics

The mean age of physicians who filled the CRF was 45.3 years; 58.6 % were male. Physicians had primary medical specialties in rheumatology (44.8 %), allergy/immunology (29.3 %) or dermatology (25.9 %), and a primary focus on adults (70.7 %) or pediatrics (29.3 %). Physicians with an adult focus were commonly rheumatologists (56.1 %) or dermatologists (26.8 %), whereas those with a primary focus on pediatrics were immunologists (29.4 %), allergists (29.4 %) or dermatologists (23.5 %). Physicians were mostly in private practice (70.7 %), and were in equal proportion from the Southern, Northeastern and Western US (29.3–31.0 %). On average, each physician had 15.1 years of experience. In the past 24 months, each physician had prescribed canakinumab to approximately 3 patients with CAPS, 2 patients with TRAPS, 2 patients with HIDS/MKD, 3 patients with FMF, and 3 patients with mixed PFS. More than half of the physicians stated they used the following resources to make diagnosis- and treatment-related decisions while treating the patients with PFS: Up to Date, peer-reviewed articles, or National Institutes of Health (NIH) Genetic and Rare Disease Information Center guidelines. Other resources were used by less than half the physicians, including textbooks, disease-specific guidelines, and institution-specific guidelines (Supplementary Table S1).

### PFS diagnosis and patient characteristics at canakinumab initiation

Patients were diagnosed with CAPS (36.7 %), TRAPS (26.5 %), FMF (26.5 %), HIDS/MKD (6.8 %), and mixed PFS (3.4 %). Among CAPS, patients had FCAS (15.0 %), MWS (16.3 %) and mixed CAPS phenotype (5.4 %). PFS was diagnosed by rheumatologists in 59.5 % of adults and 41.2 % of children. Mean (SD) age at PFS diagnosis was 6.9 (4.0) years for children and 24.8 (16.1) years for adults. The mean (SD) duration of disease from first diagnosis to canakinumab initiation was 3.0 (2.5) years for children and 7.1 (7.6) years for adults. The assessment of clinical manifestations and complications (87.1 %), age of onset (62.6 %) and family history/ancestry (57.1 %) were the commonly used methods for diagnosis. Genetic tests were used in 44.2 % of patients and were more common among children versus adults (52.9 % vs. 36.7 %). By PFS subtypes, the use of genetic testing was highest in FMF (59.5 %) and lowest in TRAPS (25.0 %). Notably, several physicians used a combination of methods to increase the certainty of the diagnosis. Of the total 147 medical charts, > 1 method of diagnosis was used in 87.1 % of patients and > 2 methods of diagnosis in 73.5 %. Per PFS subtypes, the use of > 1 and > 2 methods of diagnosis was greatest for the diagnosis of FMF, followed by CAPS (Tables 1 and 2).

**Table 1:** Patient demographics, disease characteristics and clinical manifestations of PFS patients at canakinumab initiation

Characteristics	Overall (***N***=147)	Children (***N***=68)	Adults (***N***=79)	PFS subtypes			
				CAPS (***N***=59)	TRAPS (***N***=40)	HIDS/MKD (***N***=11)	FMF (***N***=42)
**Age (years), mean (SD)**	21.7 (16.1)	9.9 (4.5)	31.9 (15.6)	24.0 (18)	21.4 (14.6)	20.6 (9.8)	18.2 (12.4)
**Female, n (%)**	63 (42.9)	28 (41.2)	35 (44.3)	23 (39.0)	18 (45.0)	6 (54.5)	19 (45.2)
**Body mass-index (for adults only; kg/m**^**2**^**), mean (SD)**	24.9 (5.6) [*N*=62]	-	24.9 (5.6) [*N*=62]	27.3 (5.9) [*N*=25]	24.0 (6) [*N*=19]	25.0 ± 4.8 [*N*=3]	22.1 ± 2.9 [*N*=16]
**Race/ethnicity, n (%)**							
White/Non-Hispanic	103 (70.1)	40 (58.8)	63 (79.7)	46 (78.0)	29 (72.5)	7 (63.6)	25 (59.5)
Hispanic	23 (15.6)	11 (16.2)	12 (15.2)	10 (16.9)	6 (15.0)	2 (18.2)	6 (14.3)
Asian/Pacific Islander	8 (5.4)	7 (10.3)	1 (1.3)	1 (1.7)	1 (2.5)	2 (18.2)	4 (9.5)
Black/Non-Hispanic	7 (4.8)	6 (8.8)	1 (1.3)	2 (3.4)	4 (10.0)	0 (0.0)	1 (2.4)
Other	6 (4.1)	4 (5.9)	2 (2.5)	0 (0.0)	0 (0.0)	0 (0.0)	6 (14.3)
**Insurance type, n (%)**							
Commercial/private	127 (86.4)	59 (86.8)	68 (86.1)	51 (86.4)	38 (95.0)	9 (81.8)	32 (76.2)
Medicare	15 (10.2)	8 (11.8)	7 (8.9)	4 (6.8)	2 (5.0)	2 (18.2)	8 (19.0)
Medicaid	7 (4.8)	3 (4.4)	4 (5.1)	4 (6.8)	2 (5.0)	1 (9.1)	1 (2.4)
Military	2 (1.4)	2 (2.9)	0 (0.0)	1 (1.7)	0 (0.0)	0 (0.0)	2 (4.8)
Other (i.e., Tricare)	1 (0.7)	0 (0.0)	1 (1.3)	1 (1.7)	0 (0.0)	0 (0.0)	0 (0.0)
**Primary medical specialty of the physician who prescribed canakinumab, n (%)**							
Rheumatology	48 (32.7)	21 (30.9)	27 (34.2)	21 (35.6)	10 (25.0)	3 (27.3)	14 (33.3)
Dermatology	46 (31.3)	23 (33.8)	23 (29.1)	18 (30.5)	14 (35.0)	5 (45.5)	11 (26.2)
Immunology	31 (21.1)	18 (26.5)	13 (16.5)	14 (23.7)	3 (7.5)	3 (27.3)	13 (31.0)
Allergy	22 (15.0)	6 (8.8)	16 (20.3)	6 (10.2)	13 (32.5)	0 (0.0)	4 (9.5)
**Primary subspecialty of the physician who prescribed canakinumab, n (%)**							
Adult	79 (53.7)	23 (33.8)	56 (70.9)	33 (55.9)	22 (55.0)	3 (27.3)	25 (59.5)
Pediatrics	68 (46.3)	45 (66.2)	23 (29.1)	26 (44.1)	18 (45.0)	8 (72.7)	17 (40.5)
**Disease duration (years) from first diagnosis to canakinumab initiation, mean (SD)**	5.2 (6.2)	3.0 (2.5)	7.1 (7.6)	6.3 (6.7)	3.9 (4.2)	6.7 (8.5)	4.7 (5.9)
**PFS severity, n (%)**							
Mild	33 (22.4)	20 (29.4)	13 (16.5)	10 (16.9)	12 (30)	5 (45.5)	6 (14.3)
Moderate	102 (69.4)	45 (66.2)	57 (72.2)	44 (74.6)	25 (62.5)	6 (54.5)	31 (73.8)
Severe	12 (8.2)	3 (4.4)	9 (11.4)	5 (8.5)	3 (7.5)	0 (0.0)	5 (11.9)
**Number of attacks/flares per year, n (%)**							
<1	7 (4.8)	5 (7.4)	2 (2.5)	5 (8.5)	0 (0.0)	0 (0.0)	2 (4.8)
1-3	50 (34.0)	19 (27.9)	31 (39.2)	20 (33.9)	18 (45.0)	4 (36.4)	8 (19.0)
4-6	64 (43.5)	29 (42.6)	35 (44.3)	22 (37.3)	15 (37.5)	6 (54.5)	24 (57.1)
7-12	20 (13.6)	11 (16.2)	9 (11.4)	8 (13.6)	7 (17.5)	1 (9.1)	5 (11.9)
>12	4 (2.7)	2 (2.9)	2 (2.5)	2 (3.4)	0 (0.0)	0 (0.0)	3 (7.1)
Unknown	2 (1.4)	2 (2.9)	0 (0.0)	2 (3.4)	0 (0.0)	0 (0.0)	0 (0.0)
**Average duration of attacks/flares, n (%)**							
<1 day	14 (9.5)	7 (10.3)	7 (8.9)	4 (6.8)	4 (10.0)	3 (27.3)	3 (7.1)
1-3 days	62 (42.2)	29 (42.6)	33 (41.8)	29 (49.2)	18 (45.0)	2 (18.2)	16 (38.1)
4-7 days	54 (36.7)	23 (33.8)	31 (39.2)	18 (30.5)	12 (30.0)	6 (54.5)	20 (47.6)
>7 days	10 (6.8)	3 (4.4)	7 (8.9)	3 (5.1)	6 (15.0)	0 (0.0)	1 (2.4)
Unknown	7 (4.8)	6 (8.8)	1 (1.3)	5 (8.5)	0 (0.0)	0 (0.0)	2 (4.8)
**PFS clinical manifestations at canakinumab initiation, n (%)**							
Fever	116 (78.9)	57 (83.8)	59 (74.7)	41 (69.5)	33 (82.5)	8 (72.7)	38 (90.5)
Fatigue/malaise	84 (57.1)	36 (52.9)	48 (60.8)	30 (50.8)	20 (50.0)	8 (72.7)	28 (66.7)
Skin/cutaneous	84 (57.1)	36 (52.9)	48 (60.8)	30 (50.8)	20 (50.0)	8 (72.7)	28 (66.7)
Musculoskeletal	74 (50.3)	31 (45.6)	43 (54.4)	27 (45.8)	19 (47.5)	6 (54.5)	26 (61.9)
Gastrointestinal	42 (28.6)	22 (32.4)	20 (25.3)	12 (20.3)	9 (22.5)	6 (54.5)	16 (38.1)
Mood/behavior	25 (17.0)	12 (17.6)	13 (16.5)	13 (22.0)	7 (17.5)	0 (0.0)	6 (14.3)
Neurological	9 (6.1)	6 (8.8)	3 (3.8)	4 (6.8)	2 (5.0)	0 (0.0)	3 (7.1)
Ocular	7 (4.8)	2 (2.9)	5 (6.3)	4 (6.8)	1 (2.5)	1 (9.1)	1 (2.4)
Lymphoid organs	5 (3.4)	1 (1.5)	4 (5.1)	2 (3.4)	0 (0.0)	2 (18.2)	1 (2.4)
Cardiorespiratory/circulatory organ	4 (2.7)	2 (2.9)	2 (2.5)	2 (3.4)	1 (2.5)	0 (0.0)	1 (2.4)
Genitourinary	3 (2.0)	1 (1.5)	2 (2.5)	2 (3.4)	0 (0.0)	0 (0.0)	1 (2.4)
Complications of PFS	1 (0.7)	1 (1.5)	0 (0.0)	1 (1.7)	0 (0.0)	0 (0.0)	0 (0.0)
Other^a^	1 (0.7)	1 (1.5)	0 (0.0)	1 (1.7)	0 (0.0)	0 (0.0)	0 (0.0)
None of the above	12 (8.2)	7 (10.3)	5 (6.3)	6 (10.2)	4 (10.0)	0 (0.0)	2 (4.8)

*CAPS* cryopyrin-associated periodic syndromes, *FMF* familial Mediterranean fever, *HIDS* hyperimmunoglobulin D syndrome, *MKD* mevalonate kinase deficiency, *PFS* periodic fever syndrome, *SD* standard deviation, *TRAPS* tumor necrosis factor receptor-associated periodic syndrome

^a^Other types of clinical manifestations included ‘hearing loss’

**Table 2:** PFS diagnosis information

	Overall (*N*=147)	Children (*N*=68)	Adults (*N*=79)	PFS subtypes			
				CAPS (*n*=59)	TRAPS (*n*=40)	HIDS/MKD (*n*=12)	FMF (*n*=42)
**PFS subtype, n (%)**							
CAPS	54 (36.7)	23 (33.8)	31 (39.2)	54 (91.5)	0 (0.0)	0 (0.0)	0 (0.0)
FCAS	22 (15.0)	8 (11.8)	14 (17.7)	22 (37.3)	0 (0.0)	0 (0.0)	0 (0.0)
MWS	24 (16.3)	11 (16.2)	13 (16.5)	24 (40.7)	0 (0.0)	0 (0.0)	0 (0.0)
Mixed CAPS phenotype	8 (5.4)	4 (5.9)	4 (5.1)	8 (13.6)	0 (0.0)	0 (0.0)	0 (0.0)
TRAPS	39 (26.5)	17 (25.0)	22 (27.8)	0 (0.0)	39 (97.5)	0 (0.0)	0 (0.0)
HIDS/MKD	10 (6.8)	6 (8.8)	4 (5.1)	0 (0.0)	0 (0.0)	10 (90.9)	0 (0.0)
FMF	39 (26.5)	19 (27.9)	20 (25.3)	0 (0.0)	0 (0.0)	0 (0.0)	39 (92.9)
Mixed PFS^a^	5 (3.4)	3 (4.4)	2 (2.5)	5 (8.5)	1 (2.5)	1 (9.1)	3 (7.1)
**Age at PFS diagnosis, mean (SD)**	16.5 (15.0)	6.9 (4.0)	24.8 (16.1)	17.7 (16.7)	17.6 (14.7)	13.9 (15.2)	13.5 (11.9)
**Time elapsed between initial symptoms and diagnosis, n (%)**							
<6 months	10 (6.8)	6 (8.8)	4 (5.1)	5 (8.5)	1 (2.5)	0 (0.0)	4 (9.5)
6–12 months	50 (34.0)	27 (39.7)	23 (29.1)	24 (40.7)	14 (35.0)	1 (9.1)	11 (26.2)
1–2 years	52 (35.4)	29 (42.6)	23 (29.1)	22 (37.3)	15 (37.5)	6 (54.5)	13 (31.0)
2–5 years	19 (12.9)	5 (7.4)	14 (17.7)	3 (5.1)	6 (15.0)	3 (27.3)	8 (19.0)
>5 years	13 (8.8)	0 (0.0)	13 (16.5)	5 (8.5)	2 (5.0)	1 (9.1)	5 (11.9)
Unknown	3 (2.0)	1 (1.5)	2 (2.5)	0 (0.0)	2 (5.0)	0 (0.0)	1 (2.4)
**Primary specialty of physician who has first diagnosed PFS, n (%)**							
Rheumatology	75 (51.0)	28 (41.2)	47 (59.5)	24 (40.7)	24 (60.0)	5 (45.5)	24 (57.1)
Immunology	29 (19.7)	20 (29.4)	9 (11.4)	18 (30.5)	3 (7.5)	0 (0.0)	10 (23.8)
Internal medicine	15 (10.2)	8 (11.8)	7 (8.9)	3 (5.1)	8 (20.0)	2 (18.2)	2 (4.8)
Allergy	15 (10.2)	5 (7.4)	10 (12.7)	9 (15.3)	3 (7.5)	2 (18.2)	2 (4.8)
Dermatology	9 (6.1)	5 (7.4)	4 (5.1)	4 (6.8)	2 (5.0)	1 (9.1)	2 (4.8)
Other^b^	1 (0.7)	1 (1.5)	0 (0.0)	1 (1.7)	0 (0.0)	0 (0.0)	0 (0.0)
Unknown	3 (2.0)	1 (1.5)	2 (2.5)	0 (0.0)	0 (0.0)	1 (9.1)	2 (4.8)
**Methods of diagnosis, n (%)**							
Assessment of clinical manifestations and complications (e.g., recurrent fever)	128 (87.1)	59 (86.8)	69 (87.3)	51 (86.4)	35 (87.5)	7 (63.6)	39 (92.9)
Age of onset	92 (62.6)	43 (63.2)	49 (62.0)	37 (62.7)	25 (62.5)	8 (72.7)	26 (61.9)
Assessment of family history/ancestry	84 (57.1)	43 (63.2)	41 (51.9)	38 (64.4)	21 (52.5)	6 (54.5)	22 (52.4)
Exclusion/rule-out diagnostics (e.g., infection, neoplasms)	77 (52.4)	36 (52.9)	41 (51.9)	31 (52.5)	17 (42.5)	4 (36.4)	28 (66.7)
Assessment of triggers (e.g., menstruation, vaccination, stress, cold, infection)	76 (51.7)	39 (57.4)	37 (46.8)	32 (54.2)	18 (45.0)	6 (54.5)	22 (52.4)
Genetic tests	65 (44.2)	36 (52.9)	29 (36.7)	27 (45.8)	10 (25.0)	6 (54.5)	25 (59.5)
Laboratory assessments (e.g., CRP, ESR, SAA)	63 (42.9)	31 (45.6)	32 (40.5)	24 (40.7)	13 (32.5)	5 (45.5)	23 (54.8)
Response to trial therapy	42 (28.6)	23 (33.8)	19 (24.1)	17 (28.8)	4 (10.0)	2 (18.2)	22 (52.4)
Other^c^	1 (0.7)	1 (1.5)	0 (0.0)	1 (1.7)	0 (0.0)	0 (0.0)	0 (0.0)
**Combination of methods used for diagnosis, n (%)**							
>1 diagnosis method	128 (87.1)	63 (92.6)	65 (82.3)	52 (88.1)	33 (82.5)	9 (81.8)	38 (90.5)
>2 diagnosis method	108 (73.5)	54 (79.4)	54 (68.4)	44 (74.6)	25 (62.5)	7 (63.6)	36 (85.7)
**Diagnoses ruled out prior to the confirmed diagnosis, n (%)**							
Fever of unknown origin	103 (70.1)	50 (73.5)	53 (67.1)	41 (69.5)	26 (65.0)	8 (72.7)	32 (76.2)
Urticaria or rash/allergy	80 (54.4)	34 (50.0)	46 (58.2)	32 (54.2)	20 (50.0)	6 (54.5)	25 (59.5)
Recurrent infection	75 (51.0)	41 (60.3)	34 (43.0)	26 (44.1)	19 (47.5)	4 (36.4)	28 (66.7)
Systemic lupus erythematosus	36 (24.5)	18 (26.5)	18 (22.8)	13 (22.0)	9 (22.5)	2 (18.2)	12 (28.6)
Vasculitis (e.g., polyarthritis nodosa, Behcet’s disease)	47 (32.0)	22 (32.4)	25 (31.6)	20 (33.9)	10 (25.0)	4 (36.4)	14 (33.3)
Pharyngitis	43 (29.3)	19 (27.9)	24 (30.4)	22 (37.3)	9 (22.5)	2 (18.2)	12 (28.6)
Rheumatoid arthritis	32 (21.8)	11 (16.2)	21 (26.6)	12 (20.3)	11 (27.5)	2 (18.2)	8 (19.0)
Neoplasms	35 (23.8)	17 (25.0)	18 (22.8)	15 (25.4)	9 (22.5)	1 (9.1)	10 (23.8)
Other juvenile idiopathic arthritis	25 (17.0)	18 (26.5)	7 (8.9)	10 (16.9)	7 (17.5)	1 (9.1)	7 (16.7)
Inflammatory bowel disease	21 (14.3)	9 (13.2)	12 (15.2)	7 (11.9)	3 (7.5)	4 (36.4)	7 (16.7)
PFS subtype other than the final diagnosis	12 (8.2)	10 (14.7)	2 (2.5)	7 (11.9)	1 (2.5)	0 (0.0)	4 (9.5)
No rule-out diagnosis	9 (6.1)	3 (4.4)	6 (7.6)	1 (1.7)	8 (20.0)	1 (9.1)	0 (0.0)
Other^d^	5 (3.4)	1 (1.5)	4 (5.1)	1 (1.7)	1 (2.5)	0 (0.0)	3 (7.1)

*CAPS* cryopyrin-associated periodic syndromes, *CRP* C-reactive protein, *ESR* erythrocyte sedimentation rate, *FMF* familial Mediterranean fever, *HIDS* hyperimmunoglobulin D syndrome, *MKD* mevalonate kinase deficiency, *PFS* periodic fever syndrome, *SAA* serum amyloid A, *SD* standard deviation, *TRAPS* tumor necrosis factor receptor-associated periodic syndrome

^a^The mixed PFS were NOMID/CINCA + HIDS/MKD, FCAS+FMF, FCAS+FMF, MWS+TRAPS, MWS+FMF

^b^Other specialties of diagnosing physician's included ‘pediatrics’

^c^Other methods of diagnosis included ‘hearing loss’

^d^Other ruled-out diagnosis included ‘scrotal pain’, ‘fatigue’, ‘arthralgia’ (2 respondents), and ‘genetic eye condition’

At the time of canakinumab initiation, the mean age of children and adults was 9.9 and 31.9 years, respectively. Fever (78.9 %), fatigue/malaise (57.1 %), skin/cutaneous (57.1 %) and musculoskeletal manifestations (50.3 %) were the most common signs/symptoms at canakinumab initiation. While fever (83.8 % vs. 74.7 %) and skin/cutaneous signs/symptoms (58.8 % vs. 55.7 %) were more common in children compared with adults, fatigue/malaise (52.9 % vs. 60.8 %) and musculoskeletal manifestations (45.6 % vs. 54.4 %) were more prevalent in adults. Overall, 43.5 % of patients experienced 4–6 attacks/flares per year, with a duration of attack being 1–3 days (42.2 %) or 4–7 days (36.7 %). These characteristics were similar in children and adults (Table 1).

By PFS subtypes, the clinical manifestation of fever was most common in FMF (90.5 %) and least common in CAPS (69.5 %). Skin/cutaneous manifestations were highest in FMF (66.7 %) and lowest in TRAPS (42.5 %). Patients who experienced 4–6 attacks/flares per year were highest in FMF (57.1 %) and lowest in CAPS (37.3 %). Notably, TRAPS had attacks/flares that for a minority of patients lasted the longest (i.e. >7 days) compared to the other subtypes of patients (Table 1).

### PFS treatment history prior to canakinumab

Among 94 patients (63.9 %) with a known first long-term treatment, the most common agents received were OCS (42.2 %), NSAIDs (41.5 %), colchicine (38.1 %), and methotrexate (21.8 %). Children more frequently received NSAIDs (47.1 % vs. 36.7 %) and colchicine (47.1 % vs. 30.4 %), while adults more commonly received methotrexate (8.8 % vs. 32.9 %). Anakinra (17.7 %) and adalimumab (11.6 %) were also used, with anakinra being used more commonly in children than adults (23.5 % vs. 12.7 %). Per PFS subtypes, the use of OCS and methotrexate was highest in patients with TRAPS (52.5 and 32.5 %, respectively) and minimum in CAPS (37.3 and 15.3 %, respectively). Colchicine was highest in FMF (61.9 %) and lowest in HIDS/MKD (18.2 %). The use of anakinra was highest in HIDS/MKD (27.3 %) while that of adalimumab was highest in TRAPS (22.5 %; Table 3).

**Table 3:** First long-term treatments among patients with PFS

	Overall (*N*=147)	Children (*N*=68)	Adults (*N*=79)	PFS subtypes			
				CAPS (*N*=59)	TRAPS (*N*=40)	HIDS/MKD (*N*=11)	FMF (*N*=42)
**Years from first long-term treatment for PFS until canakinumab initiation**							
Known, n (%)	94 (63.9)	46 (67.6)	48 (60.8)	39 (66.1)	24 (60.0)	4 (36.4)	30 (71.4)
Mean (SD)	2.5 (3.5)	1.8 (2.4)	3.1 (4.2)	2.4 (3.2)	2.6 (3.7)	3.8 (3.9)	2.6 (3.8)
**First long-term treatment for PFS** ^**a**^ **, n (%)**							
Oral corticosteroids	62 (42.2)	29 (42.6)	33 (41.8)	22 (37.3)	21 (52.5)	5 (45.5)	18 (42.9)
Nonsteroidal anti-inflammatory drugs	61 (41.5)	32 (47.1)	29 (36.7)	25 (42.4)	16 (40.0)	4 (36.4)	18 (42.9)
Colchicine	56 (38.1)	32 (47.1)	24 (30.4)	20 (33.9)	10 (25.0)	2 (18.2)	26 (61.9)
Methotrexate	32 (31.8)	6 (8.8)	26 (32.9)	9 (15.3)	13 (32.5)	3 (27.3)	7 (16.7)
Corticosteroid injection	9 (6.1)	4 (5.9)	5 (6.3)	1 (1.7)	7 (17.5)	0 (0.0)	1 (2.4)
Thalidomide	1 (0.7)	0 (0.0)	1 (1.3)	0 (0.0)	1 (2.5)	0 (0.0)	0 (0.0)
Biologics							
Anakinra	26 (17.7)	16 (23.5)	10 (12.7)	14 (23.7)	4 (10.0)	3 (27.3)	8 (19.0)
Adalimumab	17 (11.6)	5 (7.4)	12 (15.2)	6 (10.2)	9 (22.5)	1 (9.1)	2 (4.8)
Canakinumab	14 (9.5)	3 (4.4)	11 (13.9)	7 (11.9)	3 (7.5)	1 (9.1)	3 (7.1)
Rituximab	5 (3.4)	0 (0.0)	5 (6.3)	0 (0.0)	4 (10.0)	1 (9.1)	0 (0.0)
Etanercept	4 (2.7)	2 (2.9)	2 (2.5)	2 (3.4)	1 (2.5)	0 (0.0)	1 (2.4)
Infliximab	4 (2.7)	3 (4.4)	1 (1.3)	0 (0.0)	1 (2.5)	0 (0.0)	3 (7.1)
Rilonacept	2 (1.4)	2 (2.9)	0 (0.0)	2 (3.4)	0 (0.0)	0 (0.0)	0 (0.0)
Tocilizumab	0 (0.0)	0 (0.0)	0 (0.0)	0 (0.0)	0 (0.0)	0 (0.0)	0 (0.0)
Unknown	2 (1.4)	1 (1.5)	1 (1.3)	0 (0.0)	2 (5.0)	0 (0.0)	0 (0.0)

*CAPS* cryopyrin-associated periodic syndromes, *FMF* familial Mediterranean fever, *HIDS* hyperimmunoglobulin D syndrome, *MKD* mevalonate kinase deficiency, *PFS* periodic fever syndrome, *SD* standard deviation, *TRAPS* tumor necrosis factor receptor-associated periodic syndrome

^a^Treatment agents used are not mutually exclusive

Overall, NSAIDs (27.8 %), anakinra (24.1 %) and colchicine (21.8 %) were the common treatments directly preceding canakinumab. The use of NSAIDs (38.5 % vs. 17.6 %) and anakinra (33.8 % vs. 14.7 %) was more common among children versus adults. Among PFS subtypes, the use of NSAIDs was highest in HIDS/MKD (40.0 %), colchicine in FMF (35.9 %) and anakinra in CAPS (32.7 %); the use of all three agents was minimal in TRAPS (8.1-18.9 %; Table 4).

**Table 4:** Patients treated with a long-term treatment directly preceding canakinumab initiation^a,b^

Treatments directly preceding canakinumab initiation, n (%)	Overall (*N*=133)	Children (*N*=65)	Adults (*N*=68)	PFS subtypes			
				CAPS (*n*=52)	TRAPS (*n*=37)	HIDS/MKD (*n*=10)	FMF (*n*=39)
Nonsteroidal anti-inflammatory drugs	37 (27.8)	25 (38.5)	12 (17.6)	19 (36.5)	7 (18.9)	4 (40.0)	9 (23.1)
Colchicine	29 (21.8)	13 (20.0)	16 (23.5)	10 (19.2)	3 (8.1)	2 (20.0)	14 (35.9)
Oral corticosteroids	23 (17.3)	13 (20.0)	10 (14.7)	8 (15.4)	7 (18.9)	1 (10.0)	8 (20.5)
Methotrexate	19 (14.3)	3 (4.6)	16 (23.5)	5 (9.6)	8 (21.6)	0 (0.0)	6 (15.4)
Corticosteroid injection	7 (5.3)	3 (4.6)	4 (5.9)	0 (0.0)	6 (16.2)	0 (0.0)	1 (2.6)
Thalidomide	1 (0.8)	1 (1.5)	0 (0.0)	1 (1.9)	0 (0.0)	0 (0.0)	0 (0.0)
Biologics							
Anakinra	32 (24.1)	22 (33.8)	10 (14.7)	17 (32.7)	6 (16.2)	3 (30.0)	9 (23.1)
Adalimumab	12 (9.0)	3 (4.6)	9 (13.2)	4 (7.7)	5 (13.5)	1 (10.0)	3 (7.7)
Etanercept	9 (6.8)	4 (6.2)	5 (7.4)	3 (5.8)	3 (8.1)	1 (10.0)	2 (5.1)
Infliximab	6 (4.5)	4 (6.2)	2 (2.9)	1 (1.9)	3 (8.1)	1 (10.0)	1 (2.6)
Rilonacept	6 (4.5)	2 (3.1)	4 (5.9)	5 (9.6)	0 (0.0)	1 (10.0)	0 (0.0)
Rituximab	2 (1.5)	0 (0.0)	2 (2.9)	1 (1.9)	0 (0.0)	1 (10.0)	0 (0.0)
Tocilizumab	0 (0.0)	0 (0.0)	0 (0.0)	0 (0.0)	0 (0.0)	0 (0.0)	0 (0.0)
Unknown	2 (1.5)	0 (0.0)	2 (2.9)	1 (1.9)	0 (0.0)	0 (0.0)	1 (2.6)

*CAPS* cryopyrin-associated periodic syndromes, *FMF* familial Mediterranean fever, *HIDS* hyperimmunoglobulin D syndrome, *MKD* mevalonate kinase deficiency, *PFS* periodic fever syndrome, *TRAPS* tumor necrosis factor receptor-associated periodic syndrome

Notes:

^a^Excluding patients who did not receive this line of therapy, and patients who received canakinumab as first treatment

^b^Treatment agents used are not mutually exclusive

### Reasons for discontinuation of treatments prior to canakinumab

**Fig. 1:**
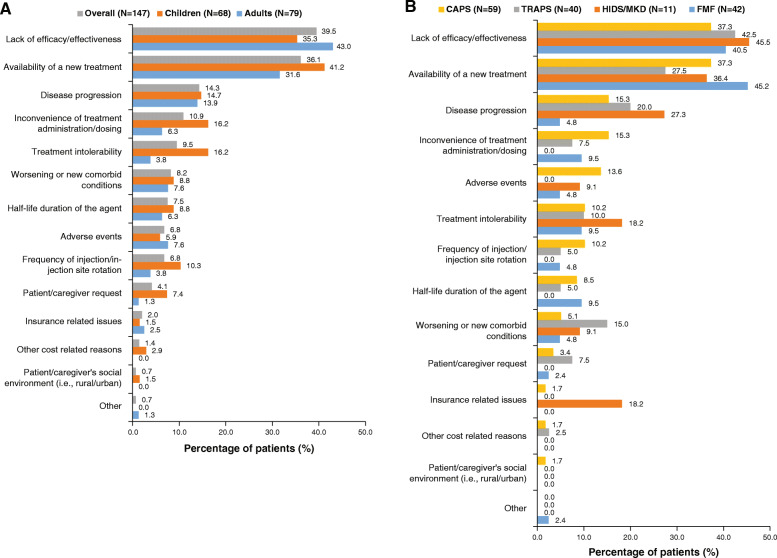
Reasons for discontinuation of treatment prior to canakinumab: **A** Children and adults. Note: More than one reason per patient possible. N is the total number of patients in the respective category. Other reasons included ‘2 biologics’. **B** PFS subtypes. Note: More than one reason per patient possible. N is the total number of patients in the respective category. Other reasons included ‘2 biologics’. CAPS: cryopyrin-associated periodic syndromes; FMF, familial Mediterranean fever; HIDS: hyperimmunoglobulin D syndrome; MKD: mevalonate kinase deficiency; PFS: periodic fever syndrome; TRAPS: tumor necrosis factor receptor-associated periodic syndrome

Among the 147 patients, the most common reasons for discontinuation of the treatment preceding canakinumab were lack of efficacy/effectiveness (39.5 %), availability of a new treatment (36.1 %) and disease progression (14.3 %). Compared to adults, the availability of a new treatment (41.2 % vs. 31.6 %), inconvenience of treatment administration/dosing (16.2 % vs. 6.3 %) and treatment intolerability (16.2 % vs. 3.8 %) were more common reasons for treatment discontinuation among children (Fig. 1A).

Per indication, the lack of efficacy/effectiveness was the highest reason for discontinuation among patients with HIDS/MKD (45.5 %) and lowest in CAPS (37.3 %). The availability of a new treatment was the most frequent reason for discontinuation in FMF (45.2 %) and least frequent in TRAPS (27.5 %), and discontinuation due to disease progression was most frequent in HIDS/MKD (27.3 %) and least frequent in FMF (4.8 %; Fig. 1B).

### Canakinumab prescribing patterns

As per the label, the recommended dose of canakinumab is 150 mg for CAPS patients with body weight > 40 kg and 2 mg/kg for CAPS patients with body weight ≥ 15 kg–≤40 kg. For children 15 to 40 kg with an inadequate response, the dose can be increased to 3 mg/kg. Administer subcutaneously every 8 weeks (q8w). For HIDS/MKD, TRAPS and FMF patients with body weight > 40 kg, the starting dose is 150 mg every 4 weeks (q4w), which can be increased to 300 mg q4w if response is inadequate. For patients with ≤ 40 kg, the starting dose is 2 mg/kg q4q, with an up titration to 300 mg q4w if response is inadequate [13]. In the current study, the median initial dose was 150 mg, irrespective of age of the patient. The average dose was 3.3 mg/kg in children and 2.3 mg/kg in adults. The frequency of canakinumab dose was q4w in 49.7 % of patients (children 52.9 %, adults 46.8 %) and q8w in 50.3 % (children 47.1 %, adults 53.2 %). By PFS subtypes, the median initial dose of canakinumab was 150 mg for all indications. The mean dose was 3.0 mg/kg in CAPS and FMF, 2.4 mg/kg in HIDS/MKD and 2.3 mg/kg in TRAPS. Canakinumab q4w dose was most common in FMF (61.9 %) and least common in CAPS (32.2 %; Table 5).

**Table 5:** Canakinumab initiation patterns among patients with PFS

	Overall (*N*=147)	Children (*N*=68)	Adults (*N*=79)	PFS subtypes			
				CAPS (***N***=59)	TRAPS (***N***=40)	HIDS/MKD (***N***=11)	FMF (***N***=42)
Age when first prescribed canakinumab (years), mean (SD)	20.7 (15.9)	9.1 (4.3)	30.7 (15.5)	22.9 (17.8)	20.4 (14.6)	19.8 (19.4)	17.4 (12.3)
Year of canakinumab initiation, n (%)							
2016	8 (5.4)	2 (2.9)	6 (7.6)	4 (6.8)	1 (2.5)	0 (0.0)	3 (7.1)
2017	58 (39.5)	27 (39.7)	31 (39.2)	26 (44.1)	15 (37.5)	5 (45.5)	15 (35.7)
2018	81 (55.1)	39 (57.4)	42 (53.2)	29 (49.2)	24 (60.0)	6 (54.5)	24 (57.1)
Canakinumab initial dose (reported by physician as mg/kg or calculated mg/kg when weight was available), mean (SD)	2.8 (1.8) [*N*=144]	3.3 (2.2) [*N*=67]	2.3 (1.1) [*N*=77]	3.0 (2.2) [*N*=57]	2.3 (0.7) [*N*=40]	2.4 (1.3) [*N*=10]	3.0 (1.8) [*N*=41]
Initial dose among physicians who reported mg/kg, mean (SD)	2.9 (1.9) [*N*=67]	2.9 (2.0) [*N*=47]	2.9 (1.5) [*N*=20]	3.3 (2.3) [*N*=27]	2.0 (0.0) [*N*=12]	2.2 (0.4) [*N*=6]	3.0 (1.7) [*N*=24]
Initial dose among physicians who reported mg	*N*=80	*N*=21	*N*=59	*N*=32	*N*=28	*N*=5	*N*=18
Mean (SD)	134.2 (46.9)	123.3 (38.1)	138.1 (49.4)	138.7 (54.0)	134.8 (39.3)	112.4 (60.2)	133.9 (38.5)
Median (Q1, Q3)	150.0 (150.0, 150.0)	150.0 (100.0, 150.0)	150.0 (150.0, 150.0)	150.0 (150.0, 150.0)	150.0 (150.0, 150.0)	150.0 (100.0, 150.0)	150.0 (150.0, 150.0)
Initial frequency, n (%)							
Every 4 weeks	73 (49.7)	36 (52.9)	37 (46.8)	19 (32.2)	23 (57.5)	6 (54.5)	26 (61.9)
Every 8 weeks	74 (50.3)	32 (47.1)	42 (53.2)	40 (67.8)	17 (42.5)	5 (45.5)	16 (38.1)
Received concomitant treatment with another long- or short-term PFS medication (including biologics), n (%)							
Yes	67 (45.6)	29 (42.6)	38 (48.1)	26 (44.1)	20 (50.0)	5 (45.5)	18 (42.9)
No	80 (54.4)	39 (57.4)	41 (51.9)	33 (55.9)	20 (50.0)	6 (54.5)	24 (57.1)

Q1, first quartile; Q3, third quartile

*CAPS* cryopyrin-associated periodic syndromes, *FMF* familial Mediterranean fever, *HIDS* hyperimmunoglobulin D syndrome, *MKD* mevalonate kinase deficiency, *PFS* periodic fever syndrome, *SD* standard deviation, *TRAPS* tumor necrosis factor receptor-associated periodic syndrome

### Reasons for canakinumab initiation

**Fig. 2:**
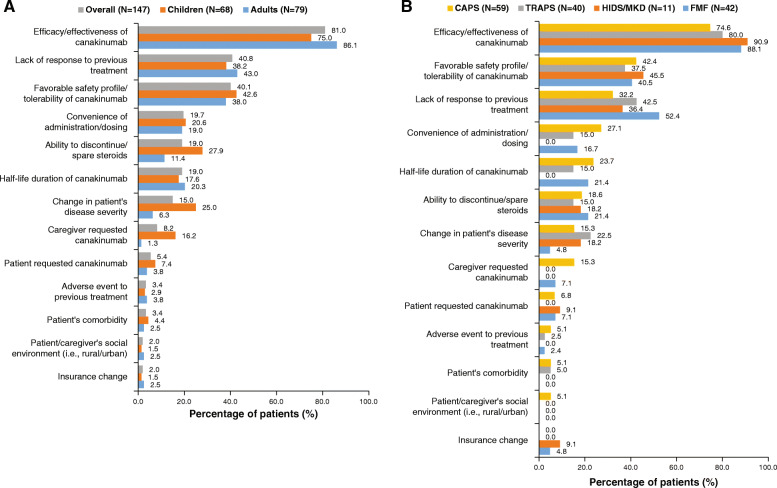
Reasons for canakinumab initiation among patients with PFS: **A** Children and adults. Note: More than one reason per patient possible. N is the total number of patients in the respective category. **B** By PFS subtypes. Note: More than one reason per patient possible. N is the total number of patients in the respective category. CAPS: cryopyrin-associated periodic syndromes; FMF, familial Mediterranean fever; HIDS: hyperimmunoglobulin D syndrome; MKD: mevalonate kinase deficiency; PFS: periodic fever syndrome; TRAPS: tumor necrosis factor receptor-associated periodic syndrome

The decision to start canakinumab was made by both physician and patient/caregiver (61 %), by physician only (35 %), and by patient/caregiver only (4 %). The most common reasons for canakinumab initiation were physician perceived efficacy/effectiveness (81.0 %), lack of response to previous treatment (40.8 %), favorable safety profile/tolerability (40.1 %) and convenience of administration/dosing (19.7 %). Compared to adults, favorable safety profile/tolerability (42.6 % vs. 38.0 %), ability to discontinue/spare steroids (27.9 % vs. 11.4 %), change in patient’s disease severity (25.0 % vs. 6.3 %) and convenience of administration/dosing (20.6 % vs. 19.0 %) were more common reasons for canakinumab initiation among children (Fig. 2A).

Per PFS subtypes, the physician perceived efficacy/effectiveness of canakinumab was the most common reason for canakinumab initiation in HIDS/MKD (90.9 %) and least common in CAPS (74.6 %), whereas lack of response to previous treatment was the most frequent reason in FMF (52.4 %) and least frequent in CAPS (32.2 %). Convenience of administration/dosing was the highest reason for canakinumab initiation in CAPS (27.1 %; Fig. 2B).

## Discussion

This retrospective medical chart review of 147 patients with PFS (54 CAPS, 10 HIDS/MKD, 39 TRAPS, 39 FMF, and 5 mixed PFS) revealed that patients commonly received NSAIDs, anakinra, OCS and colchicine as treatment options directly preceding canakinumab, which were discontinued mainly due to lack of efficacy/effectiveness, availability of a new treatment, and disease progression. The physician perceived efficacy/effectiveness of canakinumab, lack of response to previous treatment and favorable safety/tolerability were the most dominant reasons for canakinumab initiation in children and adults. To the best knowledge of the authors, this is one of the first studies providing insights on how canakinumab is initiated in US clinical practice that includes physicians’ reasons for prescribing canakinumab to PFS patients.

As expected, we observed that colchicine was often used for the first long-term treatment. However, the use of NSAIDs, OCS and methotrexate in CAPS, HIDS/MKD and TRAPS was inconsistent with a recent systematic review suggesting limited use of these agents (the review covered 72 studies of CAPS, HIDS/MKD and TRAPS published in the last two decades) [16]. Moreover, the use of OCS and NSAIDs was found to be in alignment with guidelines. The guidelines from the NIH Genetic and Rare Diseases Information Centre [17] of the US and the European League Against Rheumatism (EULAR) [11] suggest the use of NSAIDs and corticosteroids as they may provide symptomatic relief during inflammatory attacks in patients with CAPS, HIDS/MKD and TRAPS. Notably, 27 % of HIDS/MKD patients received anakinra and 23 % of TRAPS received adalimumab as the first long-term treatment, whereas up to 10 % of patients (within each PFS subtype) received canakinumab. The use of anakinra in HIDS/MKD, TRAPS and FMF, and of adalimumab across all four PFS indications was “off label”. Per the literature, anakinra has demonstrated efficacy/effectiveness in these indications, whereas the evidence on the efficacy and use of adalimumab is limited [16, 18]. Canakinumab has demonstrated efficacy/effectiveness in clinical and real world studies in all four PFS indications (CAPS, HIDS/MKD, TRAPS and FMF) [5, 15, 16, 18]. Despite being an approved treatment for the conditions hereby studied, the utilization of canakinumab as the first biologic treatment was seen less frequently than anakinra and adalimumab, and the reasons for that should be explored with further research.

This study highlighted that treatments preceding canakinumab were discontinued primarily due to the lack of efficacy/effectiveness and availability of a new treatment, with lack of efficacy/effectiveness being the most common reason for discontinuation across all four indications. Our findings about reasons for treatment discontinuation are in alignment with the literature. Multiple studies, including from the US [19], Europe [20–22] and multinational studies [23], conducted in patients with CAPS, HIDS/MKD, TRAPS and FMF have shown that lack of efficacy is the major reason for treatment discontinuation or treatment switch in these indications, followed by side effects and patient/physician preference [19–23].

This study showed that physician perceived efficacy/effectiveness of canakinumab, lack of response to previous treatment and favorable safety/tolerability were the prime reasons for canakinumab initiation in PFS patients overall. Within PFS subtypes, the lack of response to previous treatment was the highest reason for canakinumab initiation among patients with FMF (in more than half of the patients). However, the convenience of administration/dosing of canakinumab was a more common reason for patients with CAPS. Our findings about reasons for canakinumab initiation are consistent with recent literature reviews comprising evidence from 72 studies of CAPS, HIDS/MKD and TRAPS, and 38 studies of FMF with information on treatment switch to canakinumab suggesting that insufficient response/lack of effectiveness, adverse events, inconvenient dosing schedule, local injection-site reactions, or patients/physician decision were the main reasons to initiate canakinumab therapy [16, 18]. Studies have shown local reactions or pain at injection site and poor compliance as the main reasons for switching to canakinumab among patients with CAPS who were receiving another anti–IL-1 treatment [24–26].

This study also revealed notable differences between children and adults regarding reasons for previous treatments discontinuation and canakinumab initiation. The availability of a new treatment, inconvenience of treatment administration/dosing, treatment intolerability and frequency of injection/need for frequent rotation of the injection site were more prominent reasons for treatment discontinuation among children versus adults. We noted physician perceived efficacy/effectiveness of canakinumab and lack of response to previous treatment as the more common reasons for canakinumab initiation among adults than children. Nevertheless, favorable safety/tolerability, ability of canakinumab to discontinue/spare steroids and change in patients’ disease severity were more pronounced reasons for canakinumab initiation among children than adults. The greater prescription of canakinumab to children versus adults could be from the perspective of treatment compliance as pediatric patients need a less complex regimen or easier dosing schedule in order to be persistent with the treatment [24, 26]. Our findings on the treatment discontinuation and canakinumab initiation from this US study are consistent with non-US studies. Various studies, including from Europe [24, 26, 27], Turkey [25] and multinational studies [22], conducted in children with CAPS [24–26] and HIDS/MKD [22, 27] reported that inadequate efficacy, injection site local reactions/pain, poor compliance with dosing schedule of previous treatment and more convenient dosing schedule of canakinumab were the main reasons for switching to/initiating canakinumab therapy.

This study has several limitations. First, there is the potential for inaccurate data recorded in the primary charts. There is the possibility for errors introduced during data entry as physicians abstracted the information; however, logic checks were implemented to minimize these errors. This study may be affected by reporting bias (e.g., bias in favor of specific practice per guideline recommendations) or recall bias (e.g., “unknown/not sure” response options), and non-random missing data (e.g., specifically omitting a particular answer option across questions). Assessments of disease severity and treatment effectiveness in the real-world settings may be based on heterogeneous criteria. Given the small sample size, characteristics with low rates may not be estimated reliably. The large panel of physicians utilized to invite participants comprises one of the most comprehensive physician panels in the US for research purposes. However, the physicians’ response rates were low and this could introduce selection bias as the physicians who responded to the invitation to participate in the study may be different than those who did not respond. Most physicians were in private practice and this could introduce a bias to the type of patient included in this study. Although the CRF collected information on whether genetic tests were used in the diagnosis or not, it did not collect the genetic diagnosis data. The study was also not intended to collect the information on why patients did not prescribe/used canakinumab. Nonetheless, in a panel-based study, approaching physicians by email is common and this approach has resulted in a large sample given that PFS disease is rare. Furthermore, the study provided timely clinical data on patients with PFS who were treated with canakinumab. The results from this study might not be generalizable to a greater population since there were specific patient and physician inclusion criteria.

## Conclusions

In conclusion, this real-world study provided insights on the overall treatment paradigm for PFS, as well as the key drivers for initiating canakinumab in US clinical practice. The study showed that lack of efficacy/effectiveness, availability of a new treatment and disease progression are the most prominent reasons for discontinuation of treatment directly preceding canakinumab. The physician perceived efficacy/effectiveness of canakinumab, lack of response to previous treatment and favorable safety profile/tolerability were the dominant reasons for canakinumab initiation among PFS patients in both children and adults and across PFS subtypes. Notably, the favorable safety profile/tolerability of canakinumab was a more common reason for initiation among children than adults.

## Supplementary Information

**Additional file 1: Table S1. **Physician characteristics

## Data Availability

The dataset used and analyzed for this study is available upon request.

## Abbreviations

CAPS: Cryopyrin-associated periodic fever syndrome
CINCA: Chronic infantile neurological cutaneous and articular syndrome
CRF: Case report form
EULAR: The European League Against Rheumatism
FCAS: Familial cold auto-inflammatory syndrome
FMF: Familial Mediterranean fever
HIDS: Hyperimmunoglobulin D syndrome
IL-1: Interleukin 1
MKD: Mevalonate kinase deficiency
MWS: Muckle-Wells syndrome
NIH: National Institutes of Health
NOMID: Neonatal-onset multisystem inflammatory disease
NSAIDs: Non-steroidal anti-inflammatory drugs
OCS: Oral corticosteroids
PFS: Periodic fever syndromes
q4w: Every 4 weeks
q8w: Every 8 weeks
SD: Standard deviation
TNF: Tissue necrosis factor
TRAPS: TNF receptor-associated periodic fever syndrome
US: Unites States
