# Supplementary material for: Reasons for canakinumab initiation among patients with periodic fever syndromes: a retrospective medical chart review from the United States
Source: Pediatr Rheumatol Online J. 2021 Sep 14;19:143. doi: 10.1186/s12969-021-00605-2 (PMC8439059; doi:10.1186/s12969-021-00605-2)
Supplement: Supplementary file 1 — Additional file 1: Table S1. Physician characteristics [file 12969_2021_605_MOESM1_ESM.docx]

**Supplementary data**

**Table S1. Physician characteristics**

| **Physician characteristics** | **Overall**  **(N = 58)** |
| --- | --- |
|  |  |
| Age (years), mean (SD) | 45.3 (9.6) |
| Male, n (%) | 34 (58.6) |
| **Medical specialty, n (%)** |  |
| Rheumatology | 26 (44.8) |
| Dermatology | 15 (25.9) |
| Allergy | 9 (15.5) |
| Immunology | 8 (13.8) |
| **Primary subspecialty, n (%)** |  |
| Adult | 41 (70.7) |
| Pediatrics | 17 (29.3) |
| **Current Resident or Fellow, n (%)** | 4 (6.9) |
| **Type of practice, n (%)** |  |
| Private practice | 41 (70.7) |
| Solo practice | 16 (27.6) |
| Single-specialty group practice | 12 (20.7) |
| Multi-specialty group practice | 11 (19.0) |
| Hospital-owned practice | 2 (4.7) |
| Academic institution | 13 (22.4) |
| Other^†^ | 4 (6.9) |
| **Number of healthcare providers in practice, mean (SD)** |  |
| Physicians | 9.0 (14.8) |
| Nurse practitioners | 2.3 (4.2) |
| Physician assistants | 1.8 (3.7) |
| **Region of practice, n (%)** |  |
| Northeast | 18 (31.0) |
| South | 18 (31.0) |
| West | 17 (29.3) |
| Midwest | 5 (8.6) |
| **Years in practice since completion of medical school, mean (SD)** | 15.1 (8.1) |
| **Number of patients with PFS under the care of the responding**  **physician in the past 24 months, mean (SD)** |  |
| CAPS | 6.0 (8.8) |
| TRAPS | 5.3 (8.8) |
| HIDS/MKD | 5.3 (11.4) |
| FMF | 8.2 (10.9) |
| Mixed PFS | 5.8 (11.5) |
| Other PFS subtype | 0.8 (3.2) |
| **Number of patients prescribed canakinumab by the responding**  **Physician in the past 24 months, mean (SD)** |  |
| CAPS | 3.0 (4.5) |
| TRAPS | 2.4 (4.2) |
| HIDS/MKD | 2.0 (4.6) |
| FMF | 3.2 (4.7) |
| Mixed PFS | 2.9 (6.4) |
| Other PFS subtype | 0 (0.0) |
| **Number of PFS charts contributed per physician, mean (SD)** | 3.6 (3.3) |
| **Diagnosis guidelines followed by physician while treating patients with PFS, n (%)** |  |
| Up To Date | 40 (69.0) |
| Peer-reviewed articles | 39 (67.2) |
| NIH Genetic and Rare Disease Information Center | 31 (53.4) |
| Textbooks | 23 (39.7) |
| Paediatric Rheumatology International Trials Organisation (PRINTO)/Eurofever Project | 11 (19.0) |
| Institutional guidelines | 11 (19.0) |
| Specialized criteria (i.e., Livneh Tel Hashomer, Yalcinkaya-Ozen criteria) for FMF | 5 (8.6) |
| European Molecular Genetics Quality Network for Hereditary Recurrent Fevers | 4 (6.9) |
| Other^‡^ | 1 (1.7) |
| Physician does not follow any diagnosis guidelines | 2 (3.4) |
| **Treatment guidelines followed by physician while treating patients with PFS, n (%)** |  |
| Up To Date | 41 (70.7) |
| Peer-reviewed articles | 40 (69.0) |
| NIH Genetic and Rare Disease Information Center | 32 (55.2) |
| Textbooks | 24 (41.4) |
| The EULAR recommendations, endorsed by the PRES for FMF | 11 (19.0) |
| Institutional guidelines | 8 (13.8) |
| Single Hub and Access Point for Pediatric Rheumatology in Europe (SHARE) | 3 (5.2) |
| Other^#^ | 1 (1.7) |
| Physician does not refer to any treatment guidelines | 1 (1.7) |

^†^ Other types of practice settings included ‘Military hospital’, ‘Hospital’, ‘Multispecialty group’, and ‘both’.

^‡^ Other types of diagnosis guidelines referenced included ‘Google search for clinical papers’.

^#^ Other types of treatment guidelines referenced included ‘ACR’.

ACR: American College of Rheumatology; CAPS: cryopyrin-associated periodic syndromes; EULAR: European League Against Rheumatism; FMF, familial Mediterranean fever; HIDS: hyperimmunoglobulin D syndrome; MKD: mevalonate kinase deficiency; NIH: National Institutes of Health; PFS: periodic fever syndrome; PRES: Pediatric Rheumatology European Society; SD: standard deviation; TRAPS: tumor necrosis factor receptor-associated periodic syndrome.
